# Cardiomyocytes Cellular Phenotypes After Myocardial Infarction

**DOI:** 10.3389/fcvm.2021.750510

**Published:** 2021-11-08

**Authors:** Alessandra Maria Lodrini, Marie-José Goumans

**Affiliations:** Department of Cell and Chemical Biology, Leiden University Medical Center, Leiden, Netherlands

**Keywords:** myocardial infarction, apoptosis, autophagy, inflammation, senescence, dedifferentiation, cardioprotection

## Abstract

Despite the increasing success of interventional coronary reperfusion strategies, mortality related to acute myocardial infarction (MI) is still substantial. MI is defined as sudden death of myocardial tissue caused by an ischemic episode. Ischaemia leads to adverse remodelling in the affected myocardium, inducing metabolic and ionic perturbations at a single cell level, ultimately leading to cell death. The adult mammalian heart has limited regenerative capacity to replace lost cells. Identifying and enhancing physiological cardioprotective processes may be a promising therapy for patients with MI. Studies report an increasing amount of evidence stating the intricacy of the pathophysiology of the infarcted heart. Besides apoptosis, other cellular phenotypes have emerged as key players in the ischemic myocardium, in particular senescence, inflammation, and dedifferentiation. Furthermore, some cardiomyocytes in the infarct border zone uncouple from the surviving myocardium and dedifferentiate, while other cells become senescent in response to injury and start to produce a pro-inflammatory secretome. Enhancing electric coupling between cardiomyocytes in the border zone, eliminating senescent cells with senolytic compounds, and upregulating cardioprotective cellular processes like autophagy, may increase the number of functional cardiomyocytes and therefore enhance cardiac contractility. This review describes the different cellular phenotypes and pathways implicated in injury, remodelling, and regeneration of the myocardium after MI. Moreover, we discuss implications of the complex pathophysiological attributes of the infarcted heart in designing new therapeutic strategies.

## Introduction

Myocardial infarction (MI) is defined as the myocardial injury that involves cell loss due to prolonged ischaemia (1). Onset of myocardial ischaemia results from an imbalance between oxygen supply and demand of the myocardial wall, mostly due to insufficient blood flow to the tissue. The causes for the reduced blood flow to the myocardium can be many e.g., atherothrombotic coronary artery disease (CAD), coronary embolism, but also coronary spasms, sustained tachyarrhythmia, blood loss, severe anaemia or respiratory failure (2).

As early as 10 min after the onset of ischemia, ultrastructural changes like glycogen depletion, relaxed myofibrils, disruption of the sarcolemma, and mitochondrial abnormalities can be seen in the human ischemic heart and in the next few hours necrosis and apoptosis will follow (1). A strategy to reduce damage and limit infarct size is ischemic post-conditioning, brief episodes of coronary re-occlusion and reperfusion after sustained ischemia, to activate the heart's self-defence molecular programme against ischemia-reperfusion injury. The signalling pathways triggered by ischaemic conditioning are intricate and include activation of sarcolemmal receptors and cytosolic kinases, reduced mitochondrial permeability transition pore opening, calcium overload and proteolysis (3). The self-defence mechanism involves nitric oxide (NO), interleukin (IL)-10, and microRNA-144 as local triggers, while some know mediators are PKC, the RISK system, endothelial nitric oxide synthase (eNOS), and p38 (4). Despite the increasingly improved logistics of ischaemic pre- or post-conditioning, reperfusion still exacerbates the adverse changes induced by myocardial ischaemia and leading to infarction, such as rupture of mitochondria, even higher ROS levels than during ischaemia, disruption of sarcolemmal organization, and increased inflammation (3). Therefore, new approaches to confer cardioprotection, aiming to both reduce the infarct size and repair the injured myocardium, are still needed.

**Figure 1:**
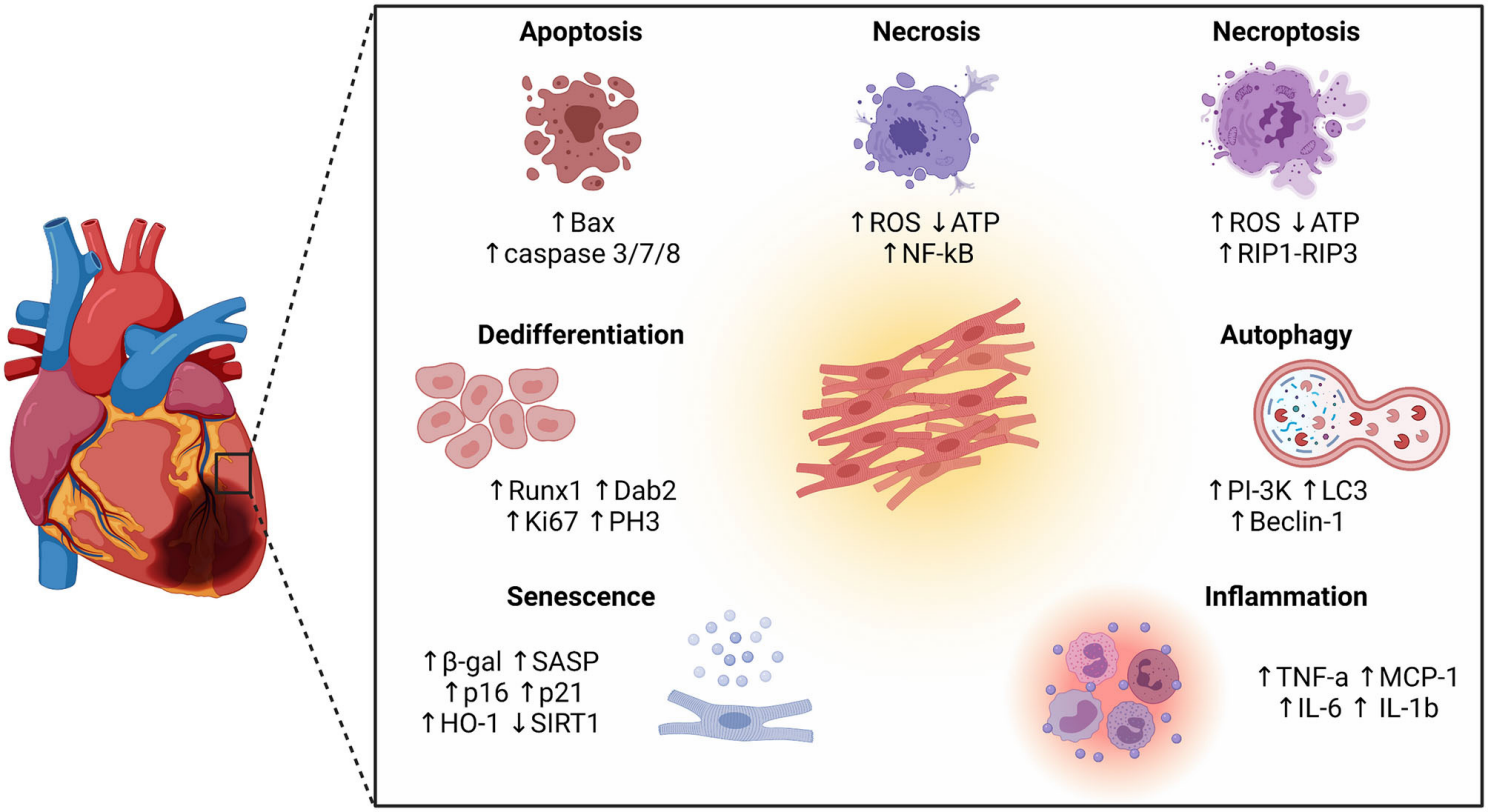
Schematic representation of different cellular phenotypes and intracellular pathways involved in the pathophysiology of the infarcted heart. Besides different forms of cell death (apoptosis, necrosis, and necroptosis), other cellular phenotypes have emerged as key players in the ischemic myocardium, including autophagy, inflammation, senescence, and dedifferentiation.

In this review we revisit the pathophysiology of MI and highlight the latest discoveries regarding cardiomyocyte cellular phenotypes and pathways implicated in cardiac injury and remodelling (Figure 1). Moreover, we discuss the complex attributes of the infarcted heart and its endogenous regenerative capacity as starting point for the design of new therapeutic strategies.

## Cell Death

MI is characterized by loss of cardiomyocytes (CMs) through different mechanisms of cell death. To develop successful treatments to protect CMs from dying, it is important to understand the different ways cells can lose viability. In general, injured cells are removed from tissue in either a programmed manner, involving a defined series of molecular events, or in an uncontrolled manner, which usually results in rupture of the cell membrane and spilling of the cellular content into the surrounding environment. The main form of controlled cell death is apoptosis, whilst the main uncontrolled form of cell death is called necrosis (5).

Apoptosis is an orderly process by which a cell dies and has its contents phagocytosed by macrophages without spilling it into the surrounding tissue. Apoptosis can be initiated *via* two pathways, the intrinsic or the extrinsic pathway. Using the intrinsic pathway, the damaged cell itself activates the apoptosis-related signaling cascades after detecting damage *via* a number of intracellular sensors, such as Puma, Noxa, and Bax. The extrinsic pathway is activated when cells of the immune system interact with specific receptors on the surface of the damaged cell known as “death receptors” (6, 7). Once one of this two pathways is initiated, the cellular autodestruction is dependent on the intracellular actions of caspases, cysteine-aspartic proteases which are key features of apoptotic cell death. Activation of the caspase-dependent signalling cascade ultimately results in DNA fragmentation by endonucleases, degradation of nuclear proteins and cytoskeleton, crosslinking of proteins, expression of ligands for phagocytic cells, and formation of apoptotic bodies. The apoptotic bodies are then phagocytosed by macrophages or the surrounding cells before they fragment (8). This results in a containment of the injury and reduces the risk of inflammation and collateral damage to the surrounding tissue.

Apoptosis is a well-described phenomenon after MI, and proposed to occur in response to oxidative stress and proinflammatory cytokines (9, 10). One week after experimentally induced MI in mice there is an increase in the number of TUNEL and cleaved caspase-3-positive nuclei (two specific markers of apoptosis). This late onset of apoptosis is then sustained during the chronic phase of ischemia and reaches maximal levels 2 weeks after left coronary artery (LCA) ligation. Of note, apoptosis after MI is mostly present in the infarct border zone and the infarct area itself, or after reperfusion in globally hypoxic zones (5, 10).

Necrosis is an uncontrolled form of cell death where the cell is damaged so severely by a sudden shock, such as an hypoxic event, that it is unable to function. The damaged cell responds by swelling, as it fails to maintain homoeostasis with its environment, and it's characterized by metabolic failure, coincident with rapid depletion of ATP, failure of ion pumps and calcium overload. Ultimately, the necrotic cell undergoes plasma membrane rupture with spillage of intracellular contents into the surrounding areas, resulting in activation of inflammation and increased tissue damage (8). Necrotic CMs and matrix fragments are highly immunogenic and cause activation of innate immune receptors and pathways, including membrane-bound toll-like receptors (TLRs), HMGB1, and RAGE. Signal transduction from these pathways converges on activation of NF-κB which promotes the production of proinflammatory cytokines and chemokines (11–13).

More recently a caspase-independent programmed form of cell death which drives myocardial remodelling has been discovered. This process is called necroptosis and it involves processes typical of both the apoptotic and necrotic pathways. For example, necroptosis is triggered by death receptors such as tumor necrosis factor receptor-1 (TNFR1) followed by the induction of the receptor-interacting protein (RIP) necroptotic complex, but it also involves production of mitochondrial ROS and depletion of cellular ATP. Activation of RIP1-RIP3 signaling results in disrupted calcium homeostasis, cell membrane rupture, and consequent cell death (14). CMs necroptosis has been described to be involved in myocardial ischemia-reperfusion injury, potentially leading to heart failure. RIP1-RIP3 expression is enhanced in mouse hearts after permanent ligation of the left anterior descending artery (LAD), while knock-out of RIP3 results in reduced inflammation and oxidative stress comparable to the levels in wild type mice (15, 16).

How and to what extent these different forms of cell death interact during ischemic injury remains unclear. Yet, targeting of either form of cell death can have an impact on the infarct size and improve cardiac function. For example cyclosporine, which inhibits apoptosis by blocking mitochondrial permeability-transition pores, can decrease the infarct size in patients with acute MI (17). Similarly, downregulation of necroptosis can protect cells against reperfusion injury. Indeed, inhibition of RIP kinase-1 *in vitro* with the compound 6E11 rescues human aortic endothelial cells from necroptosis and protects them from cold hypoxia-reoxygenation (18). Also, inhibition of necroptosis through Necrostatin-1 administration after temporary ligation of the circum?ex artery in pigs reduces infarct size, oxidative stress, and inflammatory response (19). Interestingly, animal studies have demonstrated that combined inhibition of different forms of cell death reduces the infarct size more markedly than inhibition of either type of cell death alone. In particular, simultaneous inhibition of necroptosis with Necrostatin-1 and apoptosis with Z-VAD in ischemic guinea pig hearts enhances the cardioprotective effect, resulting in improved cardiac function (20).

## Autophagy

Autophagy is a recycling and degradation mechanism essential for the quality control of intracellular proteins and organelles. In this process, cytoplasmic components are targeted and isolated from the rest of the cell within a double-membraned vesicle called autophagosome. The autophagosome then fuses with a lysosome, leading to the formation of the autolysosome. The contents of the autolysosome are eventually degraded (21). Autophagosome formation is initiated by the Class III phosphatidylinositol-3-kinase (PI-3K), Beclin-1 (also known as atg-6), and Microtubule-associated protein 1A/1B-light chain 3 (LC3) (22, 23). Control of the autophagic processes is exercised by the mammalian target of rapamycin (mToR), a serine/threonine protein kinase that can integrate information from multiple stimuli to inhibit autophagosome formation e.g., cellular nutrients, growth factors, and cellular redox state (5). Although this is a crucial process to maintain cellular homeostasis, excessive autophagy can result in overloading of cells with polyubiquitinated proteins, resulting in autophagic cell death. Indeed, autophagy is associated with neurodegenerative disorders, cancer, myopathies, and cardiomyopathies (24).

In cardiac cells, autophagy is upregulated when cells undergo stress. In experimental studies, the induction of autophagy has been observed to promote longevity, probably due to its clearance of damaged proteins and organelles (25). Conversely, the downregulation of autophagy has been observed to promote the development of hypertension, atherosclerosis, cardiac hypertrophy, ischemic heart disease and heart failure (26, 27). Autophagy has been demonstrated to have a protective role in the heart in response to ischemia by eliminating damaged mitochondria (28). Moreover, several investigators have demonstrated a dramatic increase in autophagy during the reperfusion phase of cardiac ischemia (29–33). Experiments using mouse models of MI report that autophagy occurs early after infarction and declines soon afterwards. In particular, autophagy signals increase immediately after LCA ligation in mice and are maximally expressed 1 week post ligation, in contrary to apoptosis which has a late onset. Autophagy is more prominent in the border zone of the infarct compared to remote areas and inhibition of the autophagic process by injection of a TNF-α inhibitor (CAS1049741-03-8) leads to adverse cardiac remodelling after MI, suggesting an active role of autophagy in the determination of infarct size and cardiac function (10).

Data collectively point to a mainly protective role of autophagy in the heart early after MI. Enhancing this mechanism may be a cardioprotective strategy useful to limit remodeling after ischemia.

## Inflammation

The degree of systemic and local inflammation is considered a major determinant of the extent of myocardial damage post MI and subsequent cardiac remodelling and deterioration of function. The expression of pro-inflammatory markers is elevated in the post-ischemic heart and inflammatory cells are recruited in a time and site-specific manner (34–37). Inflammation, demonstrated by an accumulation of inflammatory cells and elevated levels of TNF-α, MCP-1, IL-6, and IL-1β, reaches a peak 1 week after LCA ligation in experimental animal models, and subsides thereafter. Interestingly, 4 weeks after MI in mice the levels of inflammatory cytokines are still substantially higher compared to control, suggesting an elevated state of inflammation in the chronically ischemic heart (10). These changes in inflammatory patterns can have both protective or detrimental effects on cardiac function during chronic ischemia.

Inflammation is a key regulator for autophagy. Yuan and colleagues reported an increase in autophagy in CMs *in vitro* and *in vivo* in response to LPS or TNF-α and enhanced autophagy by rapamycin protect against LPS-mediated myocyte apoptosis (38). A dramatic increase in autophagy signals (LC3 and beclin-1) was observed soon after exposure of CMs to hypoxia or Angiotensin II. The increase in autophagy and a decline afterwards coincided with the appearance of proinflammatory signals, suggesting a close link between the two phenomena (10, 39).

Inhibition of inflammation in patients with MI and heart failure (HF) through targeting of pro-inflammatory cytokines has yielded some promising data over the last few years. In the CANTOS (Canakinumab Anti-Inflammatory Thrombosis Outcome Study) trial, more than 10,000 patients with previous MI and evidence of systemic inflammation were randomized to receive variable doses of canakinumab, a human monoclonal antibody targeting IL-1β (40). In this trial, canakinumab was found to reduce the levels of IL-1β, IL-6 and high-sensitivity C-reactive protein, while reducing the risk of secondary cardiovascular events (41). Notably, canakinumab treatment also reduces HF hospitalization and HF mortality (42).

More recently, Li and colleagues developed microparticles containing anti-IL-1β antibodies. They reported that these particles prevented cardiac remodeling and promoted cardiac repair by neutralizing IL-1β when injected in a mouse LAD ligation model (43). Similarly, Xue and colleagues investigated a potential protective effect of nanoparticles containing miR199a-3p and constructed with the membrane of macrophages which overexpress receptors for TNF-α, IL-1β and IL-6. This nanoparticle had significant anti-inflammatory activity and promoted cardiac cell survival in mice after MI. It remains unclear if the anti-inflammatory effects in this study were due to the action of miR199a-3p, the absorption of pro-inflammatory cytokines by the particle, or both (44).

Even though inflammation is recognized as critically involved in the progression of MI, the therapeutic potential of modulating this process is still under investigation. The efficacy of different anti-inflammatory treatments may vary with the type and stage of the cardiovascular disease and other patient-specific variables, indicating that further research is needed.

## Senescence

Cellular senescence refers to a state of stable cell cycle arrest in which cells become resistant to growth-promoting stimuli. Physiologically, senescence prevents the expansion of damaged cells, serving an important anti-tumorigenic function. Senescence can occur in response to a wide range of damaging stimuli, including telomere shortening, oxidative stress, and DNA damage (45, 46).

Senescent cells are characterized by typical morphological and metabolic changes, although not all biomarkers of senescence (i.e., enlarged and flattened shape, elevated senescence-associated β-galactosidase (SA-β-gal) activity, chromatin remodeling and secretion of factors that promote inflammation and tissue deterioration) are present in all senescent cells (47). Senescence is characterized by a complex phenotype and its biomarkers are not unique to senescent cells, as some markers are also observed in apoptotic cells or quiescent cells, for example. Only cells with stable cell cycle arrest which don't respond to growth factors are considered senescent (48).

Cell cycle arrest is mediated by the p53/p21^CIP1^ and p16^INK4A/pRb^ tumour suppressor pathways (49, 50). Moreover, senescent cells typically have an enlarged size and flattened shape in comparison to dividing cells and they accumulate dysfunctional mitochondria and ROS. Senescent cells show an altered lysosomal content and activity, demonstrated by increased levels of β-gal activity, which is this characteristic widely excepted as a biomarker of cellular senescence (51). Senescence can also involve a persistent DNA damage response (DDR) and accumulation of DDR-related proteins (such as γ-H2AX) in nuclear foci called DNA segments with chromatin alterations reinforcing senescence (DNA-SCARS) (52). Furthermore, many senescent cells acquire a senescence-associated secretory phenotype (SASP) that mediates non-cell autonomous effects of senescence, contributing to inflammation, and promoting tissue remodelling and repair or apoptosis (53).

Cellular senescence plays an important role in tissue remodelling both during development and in organ damage. After experimentally induced MI, ischemic injury initiates cell autophagy, apoptosis and immune-inflammatory reactions for clearance of damaged organelles and cells due to DNA damage, oxidative stress and mitochondrial dysfunction, ultimately leading to senescence in CMs. These CMs that become senescent after MI activate p16 and p53, upregulate enzyme activity of SA-β-gal and secretion of SASP, including the proinflammatory factors IL-1, IL-6 and TNF-α (54), suggesting that accumulation of senescent cells is not only a reason for organ aging, but plays also a role in the progress of myocardial damage after ischemic injury and contributes to the decrease of heart function.

Eliminating senescent cells using senolytic drugs or downregulating senescence-associated processes using cardioprotective factors could facilitate or even stimulate myocardial regeneration in the infarcted heart. Senolytic drugs are drugs targeting pro-survival pathways and proteins that are upregulated during senescence, like the BCL-2 superfamily, p53 or PI3K/AKT (55). Navitoclax, a known inhibitor of BCL-2 and BCL-xL, was the first drug in a long series of compounds able to induce selective apoptosis in a variety of cells undergoing senescence both *in vitro* and *in vivo* (56). Other more selective BCL-2 family inhibitors are currently under development as a promising strategy against senescence and aging in the heart and other tissues.

Activation of Heme oxygenase (HO) was recently reported to inhibit the activation of β-gal and to prevent CMs from developing H_2_O_2_-induced senescence (54). HO is a rapidly inducible cytoprotective factor that catalyses the oxidative cleavage of Heme into equimolar amounts of carbon monoxide (CO), iron, and biliverdin, which is then converted to bilirubin by biliverdin reductase. In the past it was already reported that the inducible form of HO (HO-1) is upregulated after ischemia/reperfusion and that HO-1 mitigates cellular injury by exerting antioxidative, anti-apoptotic and anti-inflammatory effects (57–59). Additionally, knockout of HO-1 exacerbates ischemia/reperfusion-induced myocardial injury (60, 61). These studies suggest that HO-1 is involved in cardioprotection post-MI through inhibition of CMs senescence and that enhancing HO-1 expression could improve heart function after injury.

Another factor modulated by senescence is Sirtuin-1 (SIRT1), a NAD^+^-dependent deacetylase which acts as a stress-response and survival protein (62). In mammals, SIRT1 is well-characterized to regulate several cardioprotective processes, including enhancing cell proliferation and cell survival by suppression of p53 (63, 64). Recently SIRT1 was found to be an active substrate of autophagy, which contributes to SIRT1 degradation during cellular senescence and aging (65). In ischemic conditions, SIRT1 can upregulate angiogenesis through hypoxia induced factor-1 (HIF-1), downregulate TGF-β and fibrosis, and inhibit apoptosis (64, 66). Additionally, it has been reported that Sirt1-induced p21 deacetylation can promote CMs cell cycle progression and proliferation in neonatal and adult mice, while depletion of Sirt1 reduces CMs proliferation both *in vitro* and *in vivo* (64). Of note, caloric restriction, a dietary restriction which has been described to attenuate tissue aging, promotes the activity of Sirtuins (67, 68). Furthermore, resveratrol, a polyphenol with anti-aging properties found in grapes, mimics caloric restriction by promoting FoxO and SIRT1 expression and activity (69, 70). These findings suggest that SIRT1 may be another suitable target to positively regulate cardiac protection and regeneration post-MI.

## Dedifferentiation

After MI, injured CMs within the (Nppb–positive) infarct border zone can undergo ischaemia-induced gap junctional uncoupling from their neighbours. Uncoupling can be a mechanism enacted to protect the surviving regions of the heart by reducing the spread of proarrhythmic membrane depolarization and Ca^2+^ signals from dying myocytes into the surviving myocardium. Uncoupled CMs usually undergo apoptosis over the next few days or weeks, thereby expanding the infarct zone. Recently, it has been proposed that the border zone of the infarcted myocardium is involved in postinjury cardiac regeneration. Indeed, Nppb knockout mice are unable to recover from an ischemic injury, illustrating the importance of processes that occur in the border zone for myocardial repair after MI (71).

After uncoupling from the parent myocardium, myocytes in the border zone dedifferentiate and have the ability to proliferate and redifferentiate into functional CMs, thereby becoming a potential source of newly formed contractile cells in the post-MI heart. Nppb-positive border zone CMs express dedifferentiation (e.g., Runx1 and Dab2) and proliferation (e.g., Ki67 and PH3) markers and downregulate genes implicated in cardiac muscle contraction, oxidative phosphorylation, mitochondrial activity, and fatty acid β-oxidation, all of which are highly expressed in mature CMs. Additionally, these border zone CMs switch from a MEF2 to an AP-1 (activator protein 1)–responsive gene program (71, 72). MEF2a plays a role in maintaining CM differentiation and mitochondrial activity (73), therefore the reduced accessibility of MEF2-enriched elements in border zone CMs is in line with the reduction of the mature state and mitochondrial activity of these CMs.

It has been hypothesized that enhancing the dedifferentiation process itself by activating the mitotic signalling pathways involved in embryonic heart growth could be a complementary approach for cardiac regeneration. An inhibitor of glycogen synthase kinase-3 (GSK3β), 6-Bromoindirubin-3-oxime (BIO) isolated from mollusc Tyrian purple indirubins, has been shown able to induce dedifferentiation and induce cell cycle re-entry of CMs and endothelial cells by modulating Wnt signalling (74–77). The Wnt/β-catenin pathway is involved in cardiac specification during development and there is evidence supporting a role of Wnt in response to cardiac injury (78). However, although proliferative, dedifferentiated CMs fail to efficiently induce neonatal programs for proliferation and metabolic switching to glycolysis. Additionally, in transgenic mice in which the CM cell cycle is stimulated through cardiac overexpression or inhibition of factors like cyclin D2, Tbx20, PI3K-Akt, Wnt/β-catenin, Notch, Hippo or YAP to facilitate the transition at cell-cycle checkpoints, CMs proliferate preferentially in the border zone (79–85), suggesting that the dedifferentiated state of the border zone CMs acquire after MI is necessary but not sufficient for CM renewal after injury.

Efficient redifferentiation of the dedifferentiated CMs into contractile units is necessary for the new CMs to contribute to the pump function of the heart. For successful initiation of the redifferentiation process, uncoupled CMs need to regain contact with the surviving myocardium, allowing intercellular transfer of cytoplasmic contents through gap junctions. The major isoform of gap junction proteins expressed in mammalian ventricular myocytes is Cx43. Wang and colleagues reported that CMs redifferentiation was not observed unless dedifferentiated CMs made gap junction (Cx43)–mediated cell-cell connections with neonatal ventricular myocytes in a coculture system. Additionally, they demonstrated through the use of a Cx43–small interfering RNA (siRNA), designed to inhibit Cx43 expression in CMs, reduced redifferentiation of uncoupled CMs (72). Considering these studies, targeting the dedifferentiated CMs in the infarct border zone specifically and promoting the endogenous redifferentiation process by facilitating gap junction formation may be a promising strategy for heart regeneration after MI.

## Cardioprotective Strategies Against MI

**Figure 2:**
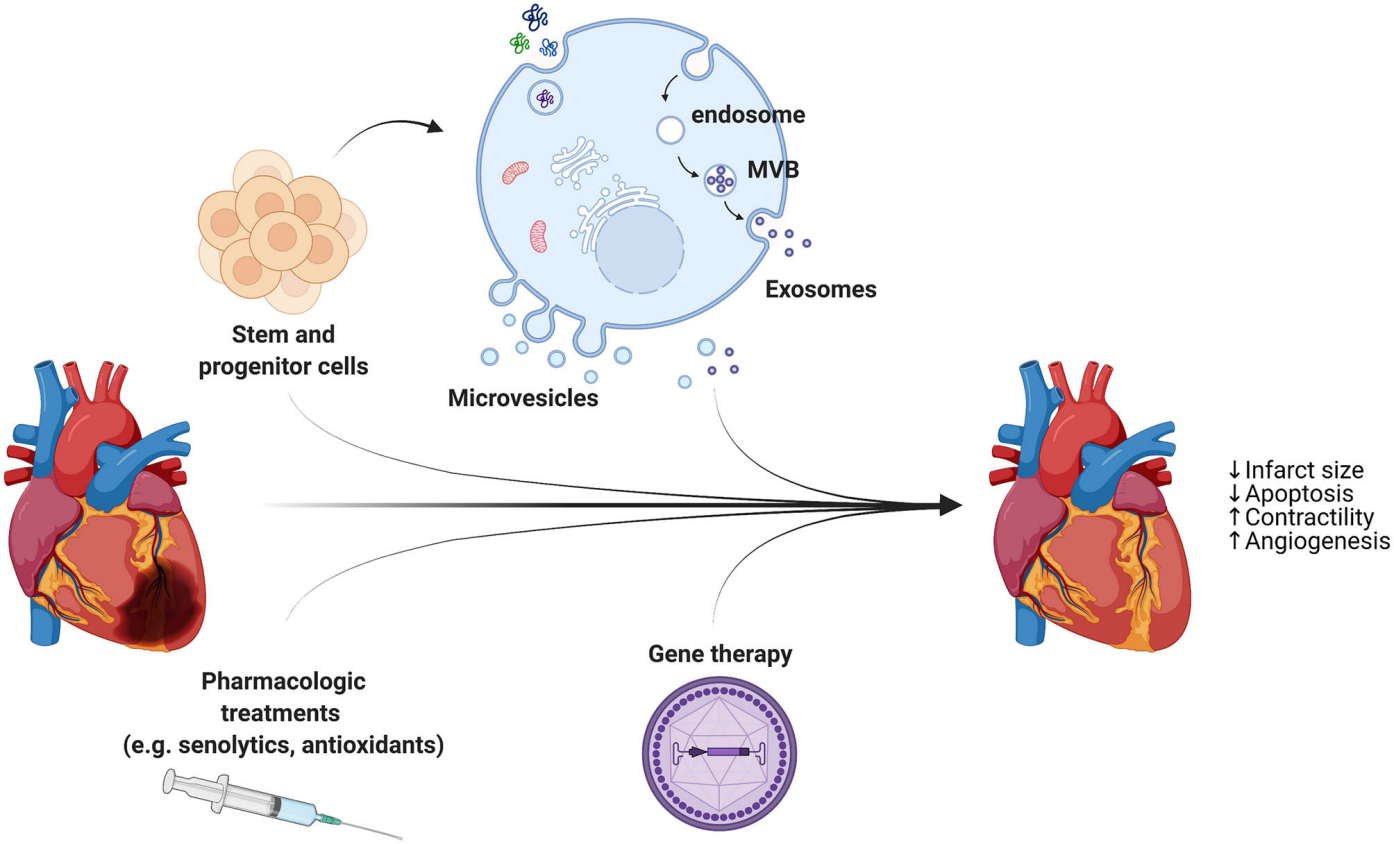
In addition to reperfusion therapy, cardioprotective strategies are necessary to reduce the infarct size and repair the injured myocardium. A combination of different approaches, including stem cell- and progenitor cell-derived extracellular vesicles, pharmacologic treatments, and AAVs for gene therapy, holds great promise for heart regeneration through limiting cell death and stimulating cardiomyocytes proliferation and blood vessel growth.

Patients suffering from cardiovascular diseases undergo treatments, surgeries and medications which alleviate symptoms and decelerate disease progression, but fail to repair damaged tissue. In animal studies, the ischemic conditioning protocols have consistently been shown to reduce infarct area and increase myocardial salvage after prolonged ischemia. However, both animal and human studies have demonstrated mixed effects of pre- and post-conditioning on ischemia or reperfusion-induced arrhythmias. Therefore, it has been proposed that cardioprotective and regenerative strategies are necessary in addition to reperfusion to reduce the infarct size and repair the injured myocardium (Figure 2).

Since the proliferating and self-healing capacity of CMs in adults is limited, the first approaches in the cardiac regenerative medicine field focused on exploiting the potential of autologous or allogeneic transplants of stem cells for heart repair. Stem cells are specified as undifferentiated cells possessing the ability to generate, sustain, and replace terminally differentiated cells *via* unlimited replication. However, the therapeutic use of pluripotent stem cells (ESCs and iPSCs), potentially able to differentiate into mesodermal-derived CMs, is still limited mainly due to the risk of immune rejection, genetic instability, teratoma risk, low induction efficiency, and ethical issues (86, 87). Additionally, several independent studies have demonstrated that, while providing significant improvement in heart function, injection of stem and progenitor cells into the damaged myocardium results only in limited differentiation, mainly into vascular lineages (88, 89).

Alternative mechanisms and explanations for the beneficial effects of stem cell transplants despite low levels of differentiation have been thoroughly investigated. Stem cell-derived paracrine effects have emerged as a very promising strategy for the reactivation of endogenous mechanisms of repair and regeneration in several disease models (90–94). In these studies, cell transplantation has been demonstrated to indirectly contribute to tissue regeneration by modulating cellular processes rather than direct differentiation into new functional tissue. Interestingly, fibroblasts engineered to resemble cardiosphere-derived cells (CDCs) by overexpression of β-catenin and Gata4, are able to improve cardiac function and mouse survival when transplanted into a model of acute MI through activation of cardioprotective signals and reduction of fibrosis in the surrounding tissue (95). These discoveries have led to a significant paradigm shift, from exploring the stem cell genome to analyzing the stem cell “secretome” as the whole of growth factors and chemo-attractant molecules produced by paracrine secretion. In the analysis of the stem cell secretome, there is growing interest on the characterization of extracellular vesicles (EVs). These EVs are membrane-bound cellular components enriched with soluble bioactive factors (e.g., proteins, lipids) and RNA (regulatory miRNAs and mRNA) eliciting wide-ranging effects while mediating horizontal inter-cellular transfer of genetic information to recipient cells and modulating their function (96). EVs are secreted as micro-sized (microvesicles, diameter 0.2–1 μm) and nanosized (exosomes, diameter 40–150 nm) particles. Microvesicles are released as shedding vesicles by direct budding of the plasma membrane, while exosomes are produced in endosomal multivesicular bodies (MVB) and secreted as the MVB fuses with the plasma membrane (97). The isolation and characterization of exosomes (Exo) is still difficult, and distinct techniques such as chromatography, centrifugation, precipitation, and affinity-isolation are used, often in combination (98–101).

Some studies suggest that the beneficial effects observed in preclinical models of ischemic heart disease following stem cell transplantation are mediated by progenitor cell-derived exosomes. These beneficial effects include the activation of pro-survival, angiogenic, anti-inflammatory and anti-fibrotic pathways, and the stimulation of resident endogenous progenitors, overall enhancing organ function (102). EVs from adult mesenchymal stem cells (cardiac progenitor cells and bone marrow) have been demonstrated to provide cardioprotection during acute myocardial infarction (90, 103–108), enhance wound healing (109), counteract graft-vs.-host-disease (GVHD) (110), reduce renal injury (111), mediate liver regeneration (112), stimulate neural plasticity following stroke (113), and counteract Doxorubicin-induced cardiotoxicity (114). Moreover, evidence suggests that EVs produced by epicardial cells are able to enhance proliferation in primary neonatal murine CMs and H9C2 cells *in vitro* and promote cell cycle re-entry when injected into the injured area of infarcted neonatal hearts (115). Since cell-free delivery of bioactive cargos by EVs recapitulates similar beneficial responses to stem cell transplantation, EVs offer more benefits over conventional cell therapy, being an immunologically unresponsive agents (116). Therefore, exosomes from stem and progenitor cells are promising candidates to promote autophagy and CMs survival, while working against senescence and SASP, but they may be engineered to guarantee substantial therapeutic effects.

Other cell-free therapies that are being investigated for their potential in mammalian heart regeneration include adeno-associated viruses (AAVs). These are non-integrating viruses and have been shown to achieve high levels of transduction in quiescent cells and CMs, with AAV1, AAV6, AAV8 and AAV9 identified as the most cardiotropic ones (117, 118). Gene therapies employing AAVs could potentially be used to jumpstart the cell cycle and promote CMs proliferation. However, it must be taken into consideration that this strategy may lead to increased cancer risk in non-cardiomyocytes. Indeed, multiple candidate genes that have been considered for this approach, including Hippo and YAP, are involved in certain types of cancer (119, 120), therefore requiring precise targeting to CMs to minimize oncogenic risks while using this approach.

## Conclusion

A combination of different approaches, including stem cell- and progenitor cell-derived Exo, AAVs for gene therapy, and specific medication (e.g., senolytic drugs), to target the border zone of the infarct holds great promise for heart regeneration through stimulating CM proliferation and redifferentiation and blood vessel growth in the damaged hearts. Yet multiple issues, like specific induction of CMs, potential cancer risk in non-cardiomyocytes, and incomplete electrical coupling between newly generated cells and host cardiac tissue still need to be fully addressed before clinically applying these strategies.

## Author Contributions

All authors listed have made a substantial, direct and intellectual contribution to the work, and approved it for publication.

## Funding

The authors of this work were supported by the Leiden University Medical Center and the REGenerative MEDicine Crossing Borders (RegMedXB) foundation.

## Conflict of Interest

The authors declare that the research was conducted in the absence of any commercial or financial relationships that could be construed as a potential conflict of interest.

## Publisher's Note

All claims expressed in this article are solely those of the authors and do not necessarily represent those of their affiliated organizations, or those of the publisher, the editors and the reviewers. Any product that may be evaluated in this article, or claim that may be made by its manufacturer, is not guaranteed or endorsed by the publisher.
